# Buoyancy control in ammonoid cephalopods refined by complex internal shell architecture

**DOI:** 10.1038/s41598-021-87379-5

**Published:** 2021-04-13

**Authors:** David J. Peterman, Kathleen A. Ritterbush, Charles N. Ciampaglio, Erynn H. Johnson, Shinya Inoue, Tomoyuki Mikami, Thomas J. Linn

**Affiliations:** 1 Department of Geology and Geophysics, University of Utah, Salt Lake City, UT 84112 USA; 2 Department of Science, Mathematics, and Engineering, Wright State University (Lake Campus), Celina, OH 45822 USA; 3 Paleontological Research Institution, Ithaca, NY 14850 USA; 4 Hokkaido University Shuma-no-kai, Hokkaido, 060-0817 Japan; 5 Department of Biological Sciences, University of Tokyo, Tokyo, 113-0032 Japan; 6 Division of Paleontology, Frontier Gateway Museum, Glendive, MT 59330 USA

**Keywords:** Evolution, Palaeontology, Ecophysiology, Evolutionary ecology, Palaeoecology

## Abstract

The internal architecture of chambered ammonoid conchs profoundly increased in complexity through geologic time, but the adaptive value of these structures is disputed. Specifically, these cephalopods developed fractal-like folds along the edges of their internal divider walls (septa). Traditionally, functional explanations for septal complexity have largely focused on biomechanical stress resistance. However, the impact of these structures on buoyancy manipulation deserves fresh scrutiny. We propose increased septal complexity conveyed comparable shifts in fluid retention capacity within each chamber. We test this interpretation by measuring the liquid retained by septa, and within entire chambers, in several 3D-printed cephalopod shell archetypes, treated with (and without) biomimetic hydrophilic coatings. Results show that surface tension regulates water retention capacity in the chambers, which positively scales with septal complexity and membrane capillarity, and negatively scales with size. A greater capacity for liquid retention in ammonoids may have improved buoyancy regulation, or compensated for mass changes during life. Increased liquid retention in our experiments demonstrate an increase in areas of greater surface tension potential, supporting improved chamber refilling. These findings support interpretations that ammonoids with complex sutures may have had more active buoyancy regulation compared to other groups of ectocochleate cephalopods. Overall, the relationship between septal complexity and liquid retention capacity through surface tension presents a robust yet simple functional explanation for the mechanisms driving this global biotic pattern.

## Introduction

Profound increases in biological complexity are easily observed in the fossil record^1–11^. Ammonoid cephalopods are among the most iconic fossils and their chambered shells present ideal targets to study the evolution of complexity through time^6,8,10–12^, due to the clade’s large temporal range (~ 340 Ma). Ammonoids also had high evolutionary rates, wide geographic distributions, high morphological disparity, and high taxonomic diversity^3,10,13,14^. Because of these properties, ammonoids are key tracers of biotic crises, and give critical ecological context to important events in evolution and extinction. From the Early Devonian to Late Cretaceous, ammonoids produced internal shell architecture of increasing complexity, eventually yielding fractal-like patterns that have captured the attention of scientists for centuries^6,12,14–18^. These intricate structures may provide evidence for the remarkable success of these animals that were major components of marine ecosystems for most of the current eon.

**Figure 1:**
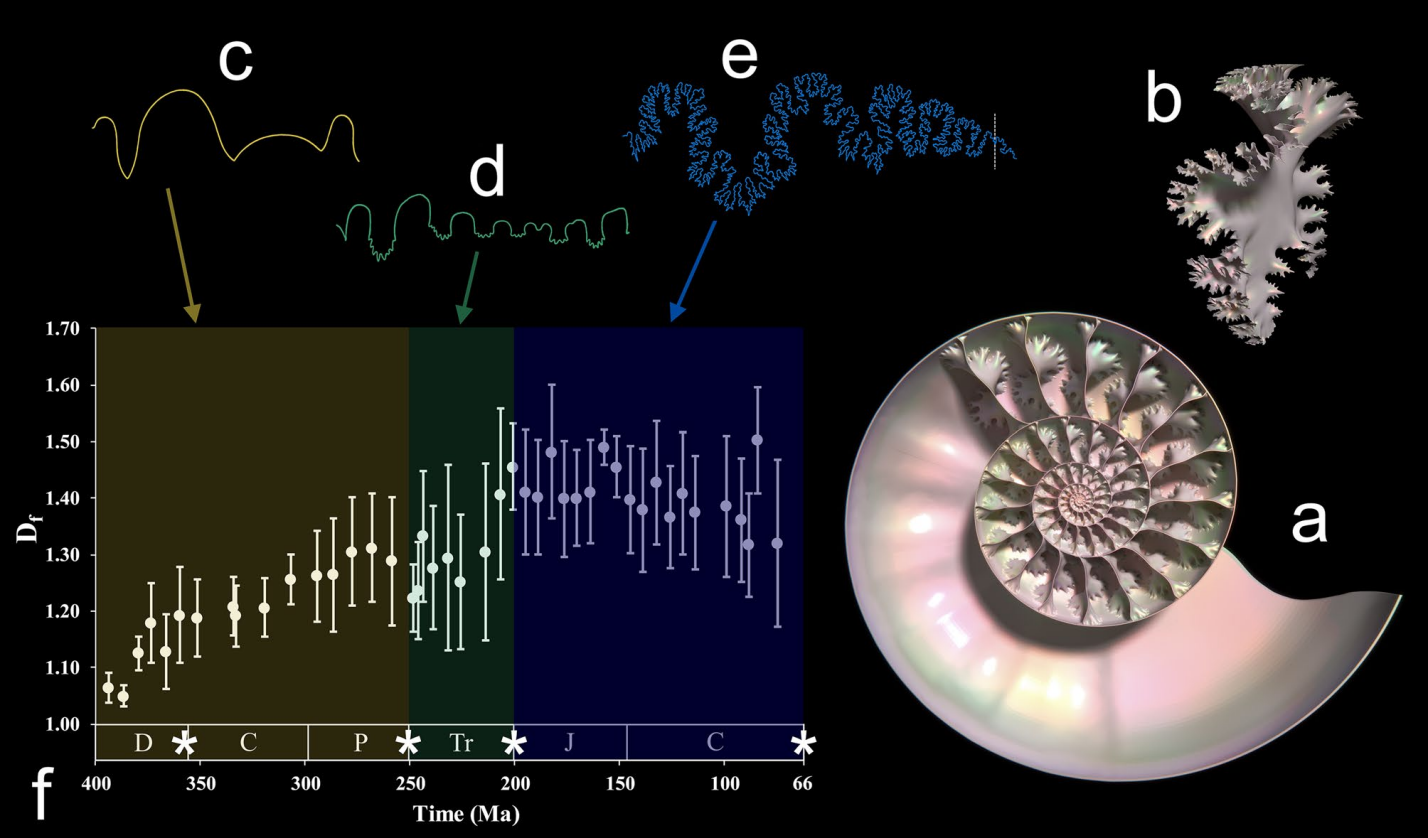
Change in ammonoid cephalopod shell anatomy through time. (**a**) Cross section of a virtually reconstructed ammonoid (*Eopachydiscus marcianus*). (**b**) Septum of the shell in (**a**) showing its frilled margins. (**c**) Goniatitic suture pattern (*Manticoceras*; modified from Arkell et al.^87^). (**d**) Ceratitic suture pattern (*Otoceras*; modified from Arkell et al.^87^). (**e**) Ammonitic suture pattern of (**a**) and (**b**). All sutures displayed are only half of the pattern (hemisutures; oriented ventral-left and anterior-upwards). (**f**) Data from Boyajian and Lutz^6^ showing increasing sutural complexity (measured by fractal dimension; D_f_) through geologic time. Global mass extinctions are indicated with stars on the timeline. The colored regions and corresponding sutures in (**c**–**e**) represent the dominant suture types during those time periods.

The conch of ectocochleate (externally-shelled) cephalopods and some early coleoids is essentially a long tube: the animal’s body residing at the growing aperture, while walling off the unoccupied rear portion into a series of chambers (camerae; Fig. 1A). As the mollusk produces a fresh wall, or septum (Fig. 1B), it drains fluid from the recently-isolated chamber to regulate buoyancy^19^. The chambers remain connected, however, by the siphuncle, an organ capable of adjusting chamber liquid through osmosis^20,21^. Furthermore, this organ can facilitate pumping against the hydrostatic pressure of water, allowing functionality at a great range of depths^22,23^. Capillary membranes (i.e., the pellicle) line the chambers^24–27^, which allow liquid to be transported to the siphuncle even when they are decoupled^19,21,26,28,29^. The suture formed at the junction between each septum and the inner surface of the shell wall presents a repeating pattern easily observed in fossils (Fig. 1A,E). Suture patterns are simple, straight or sinuous in nautiloids. This group includes the only extant ectocochleates—the nautilids. Extant *Nautilus* are used as frequent, but incomplete, models for ammonoid biology^30^. After originating from the bactritoids, which diverged from nautiloids ca. ~ 420 Ma ago^31^, ammonoids produced several derived clades with increasingly complex suture expressions: agoniatitic and goniatitic (simple sinuous, zig-zags; Fig. 1C), ceratitic (sinuous with accessory elements on the lobes; Fig. 1D), and ammonitic (several levels of accessory elements due to the fractal-like frilling of the septal margins; Fig. 1B,E). Moreover, each of these major shifts occurred between global mass extinctions: most species with goniatitic sutures went extinct during the end-Permian extinction, species with ceratitic sutures becoming dominant and going extinct at the end-Triassic mass extinction, then species with ammonitic sutures flourishing in the Jurassic and Cretaceous (Fig. 1F). This general trend towards increased septal frilling is represented by the dominant groups during these time periods. It should be noted that other suture types are less common, but present within clades that persist through these mass extinctions (e.g., Prolecanitida with goniatitic sutures in the Late Paleozoic to Triassic; Phylloceratida with ammonitic sutures originating in the Triassic and persisting until the Late Cretaceous). Furthermore, there are several examples of homeomorphs with sutures resembling other clades outside of their characteristic groups: cyclolobid and perrinitid goniatites with ammonitic sutures in the Late Paleozoic; tissotiid, engonoceratid, and sphenodiscid ammonites with ceratite-like sutures in the Cretaceous; Cretaceous flickiid ammonites with sutures resembling the agoniatitic type; and Cenozoic aturiid nautiloids with sutures resembling goniatites. All ammonoids, however, went extinct at the end of the Mesozoic, survived by the closely related coleoids (octopus, squid, etc.). Their more distant relatives, the simply-sutured nautiloids, were able to weather each mass extinction and represent the only living ectocochleates today^10,32,33^.

### Functional interpretations of septal complexity

The adaptive value of complex ammonoid septa is enigmatic, and remains a contentious issue. If complex septa lacked a primary function, they could have been artifacts of fabricational noise, resulting from the growth processes involved during septal morphogenesis^34^. Functional explanations for septal complexity range from physiological to mechanical; for a review, see Klug and Hoffmann^18^. The most popular functional interpretations of this morphology concern buttressing against hydrostatic pressure or other forms of stress^13,17,35–39^; an explanation which regularly appears in textbooks^40–43^. Recent approaches have used sophisticated modeling techniques to explore shell stress. Simulations by Lemanis^44^ demonstrate that higher-order folds, characteristic of ammonitic septa, do not reduce the tensile stress of the shell, however, increased point load resistance is suggested^45^. Johnson et al.^46^ investigated shell strength by performing physical compression experiments on 3D-printed models and found that sutural complexity does not relate to compression resistance. Furthermore, others have found that fluted septa may actually decrease certain types of stress resistance^,^^44,47^. Concerning hydrostatic pressure, fossil occurrences and lithological data do not support a relationship between increased septal complexity and deeper habitats^48–50^. Such a relationship would be expected if complex septa were adaptations to resist implosion at greater depths. Another interpretation suggests that more complex septa are more firmly secured in the shell during the mineralization process^51^.

Several other functional interpretations for complex septa focus on cameral liquid transport and buoyancy modification. Higher sutural complexity increases the surface area of each septum and therefore the surface area of the conchiolin membrane (pellicle) that lined them. This larger surface may have facilitated faster chamber emptying^52^. However, this function depends upon osmotic pumping, which is influenced by variable siphuncular anatomy^53^ and may have been limited by the osmotic gradient between the siphuncle and chamber fluids^21^. Other interpretations suggest that the marginal crenulations of septa could have retained liquid by surface tension, possibly improving buoyancy adjustment^24,25,28,47,54,55^. The retention of chamber liquid is supported by empirical assessments of ammonoid buoyancy, demonstrating that some amount of liquid is required for a near neutrally buoyant condition^56–60^. Extant *Nautilus* are slightly negatively buoyant, which is easier for the living animal to manage^21^. This similar condition may apply to ammonoids as well.

Recent advances in virtual modeling and computed tomography (CT) now allow intricate internal shell anatomy to be extracted from ammonoid fossils^56–63^. Here, we use 3D printed models of virtually reconstructed septa to investigate the outstanding hypotheses regarding cameral liquid retention capacity and surface tension.

## Results

**Figure 2:**
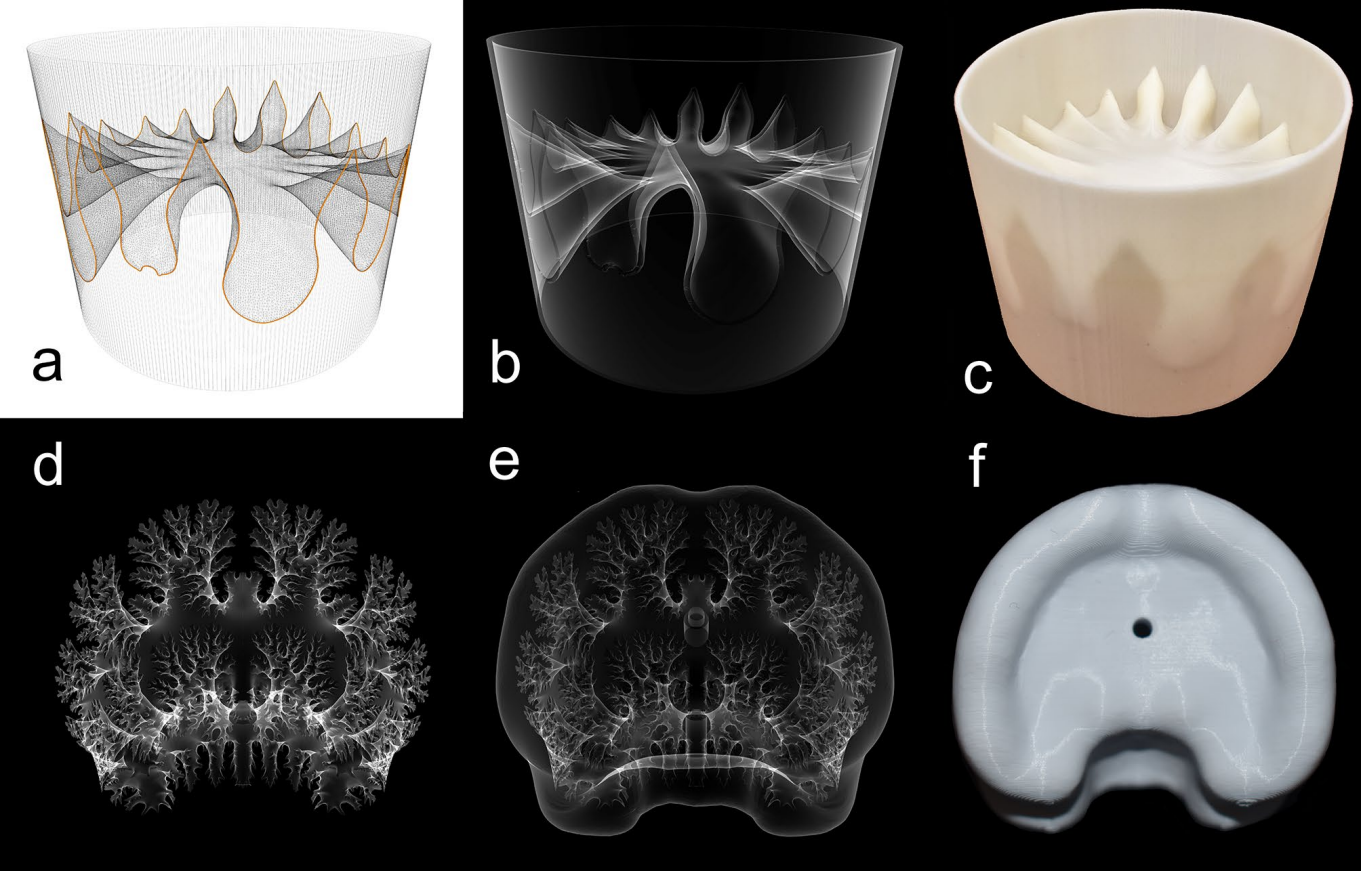
Creation of cylindrical shells models (**a**–**c**) and chamber models (**d**–**f**) used in liquid retention experiments. (**a**) Wireframe view showing the suture (orange) wrapped around the internal whorl section. (**b**) Extruded, virtual model with the cylindrical shell and septum unified together. (**c**) Final 3D printed model used to measure the liquid retained by surface tension in the septal recesses. (**d**) Virtual model of a single chamber (camera). (**e**) Virtual model of the camera subtracted from a bounding volume. (**f**) Final, 3D printed model with empty chamber inside and holes for drainage on the adoral, adapical, and ventral sides of the model.

Liquid retention was measured in idealized, 3D-printed models of single septa in cylindrical shells, and within entire chambers of more realistic models (Fig. 2). The theoretical, cylindrical shell models allow the relative differences in liquid retention to be measured from sutures that fall on a continuum of increasing complexity: nautiloid (*Orthoceras regulare*), simple goniatitic (*Manticoceras* sp.), complex goniatitic (*Schistoceras missouriense*), simple ceratitic (*Xenodiscus carbonarius*), complex ceratitic (*Otoceras woodwardi*), simple ammonitic (*Baculites compressus*) and complex ammonitic (*Diplomoceras notabile*). Here, the terms simple and complex are chosen based on the relative complexity within each suture category (number of primary elements for non-ammonitic sutures and number of higher order elements for ammonitic sutures). More realistic models were constructed from morphological data of actual specimens (*Damesites* sp. and *Menuites oralensis*) and across multiple size ranges (15, 25, 40, 60, and 90 mm). The sutures of these chamber models were iteratively smoothed to successively erase the highest order folds, producing additional sets of models. These different versions allow scaling and complexity to be investigated while holding other morphological characters that could influence liquid retention constant (e.g. whorl section shape, septal spacing, and shell sculpture). A second set of all models were treated with a thin biomimetic hydrophilic coating, similar to the membrane lining extant *Nautilus* chambers^26^ (see Supplementary Figs. 1, 2 and Supplementary Tables 11, 12).

### Liquid retention in theoretical, cylindrical models

**Figure 3:**
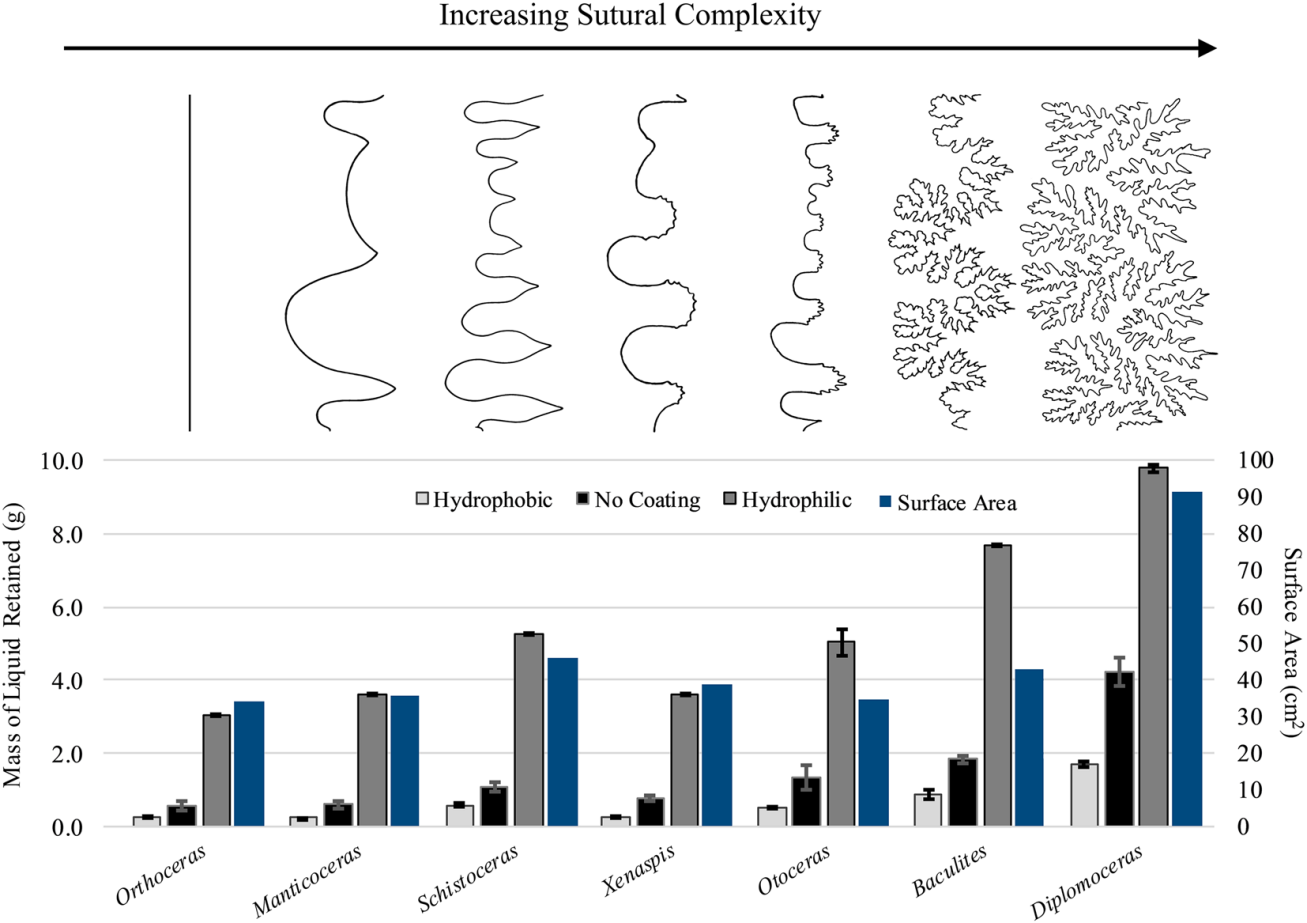
Liquid retained in the cylindrical septa models (left axis) with hydrophobic silicone coating (light grey), no coating (black), and hydrophilic micro-dispersed oxidized cellulose coating (dark grey). Each model was printed at 6 cm whorl height (dorsoventral length). The surface areas (blue) of these septa are reported on the right axis. Suture patterns from the left to the right: *Orthoceras regulare* (nautiloid), *Manticoceras* sp. (simple goniatitic), *Schistoceras missouriense* (complex goniatitic), *Xenodiscus carbonarius* (simple ceratitic), *Otoceras woodwardi* (complex ceratitic), *Baculites compressus* (simple ammonitic), and *Diplomoceras notabile* (complex ammonitic). All suture patterns shown are right hemisutures only, oriented venter-downwards, anterior-left. The nautiloid suture is approximated from Teichert et al.^85^ (pg. 225, fig. 153), the goniatitic and ceratitic sutures are modified from Arkell et al.^87^, the suture of *Baculites* is modified from Peterman and Barton^55^, and the suture of *Diplomoceras* is modified from Jones^88^. Error bars represent one standard deviation (n = 10).

When applied directly to the printed surface of ABS (acrylonitrile butadiene styrene) plastic models, liquid remained along the septal margins due to surface tension and adhesion. Maximum retention occurs when septa adjoin the shell at low angles and with more crenulations of the septal margins because the adhesive surface in relation to surface tension increases markedly. Septa that correspond to nautiloid, goniatitic, or ceratitic sutures yield little difference in absolute volume of retained liquid (Supplementary Tables 1–4). Models with ammonitic sutures, however, retain much more liquid. This difference is easily attributed to more optimal water retention in recesses along both sides of the frilled septal margins (Fig. 3).

Further results compare liquid retained in the same models, but treated with a thin hydrophilic coating (micro-dispersed oxidized cellulose) to mimic the pellicle (conchiolin membrane) of a living ectocochleate cephalopod. The applied coating looks and behaves very similar to the pellicle of extant *Nautilus* (Supplementary Fig. 2)*.* Models with this hydrophilic coating retain much more liquid than models in non-coated trials (Fig. 3). Here, additional water is retained along both the margins and centers of the septa. There is no clear directional relationship between septal surface area and gross water retention. The cylindrical septa experiments (Fig. 3) allow comparisons of the relative differences in residual liquid mass and surface area between different suture types. The model with a *Diplomoceras* suture retains about 3.2 times the mass of liquid compared to the simple nautiloid-like (*Orthoceras*) model, but only 2.7 times the septal surface area. Furthermore, the *Baculites* model retains 2.5 times more liquid while only increasing its surface area by 1.2 times when compared to the nautiloid model. These differences suggest that liquid held along the septal surfaces is important, but the shape of the septal margins is the first-order feature for liquid retention. The *Baculites compressus* model demonstrates this well (Fig. 3) because its septal surface area is not much different compared to the goniatitic or ceratitic models, but much more liquid is retained (around 1.5 to 2 times for the models with hydrophilic coatings).

The hydrophobic silicone-coated models display the same general trend as the other experiments, however, much less liquid is retained. By visual inspection, minor amounts of liquid were still held at the margins, only weakly adhering to the models. While the chambers of these cephalopods did not have this capillary property during life, the hydrophobic coating experiments demonstrate a wider range of physical conditions for comparison. During this scenario, liquid retention is governed by weak adhesion, and minor beads of liquid that failed to escape the complex folds of the ammonitic models during draining.

### Experiments on chamber models, scaling relationships, and septal complexity

**Figure 4:**
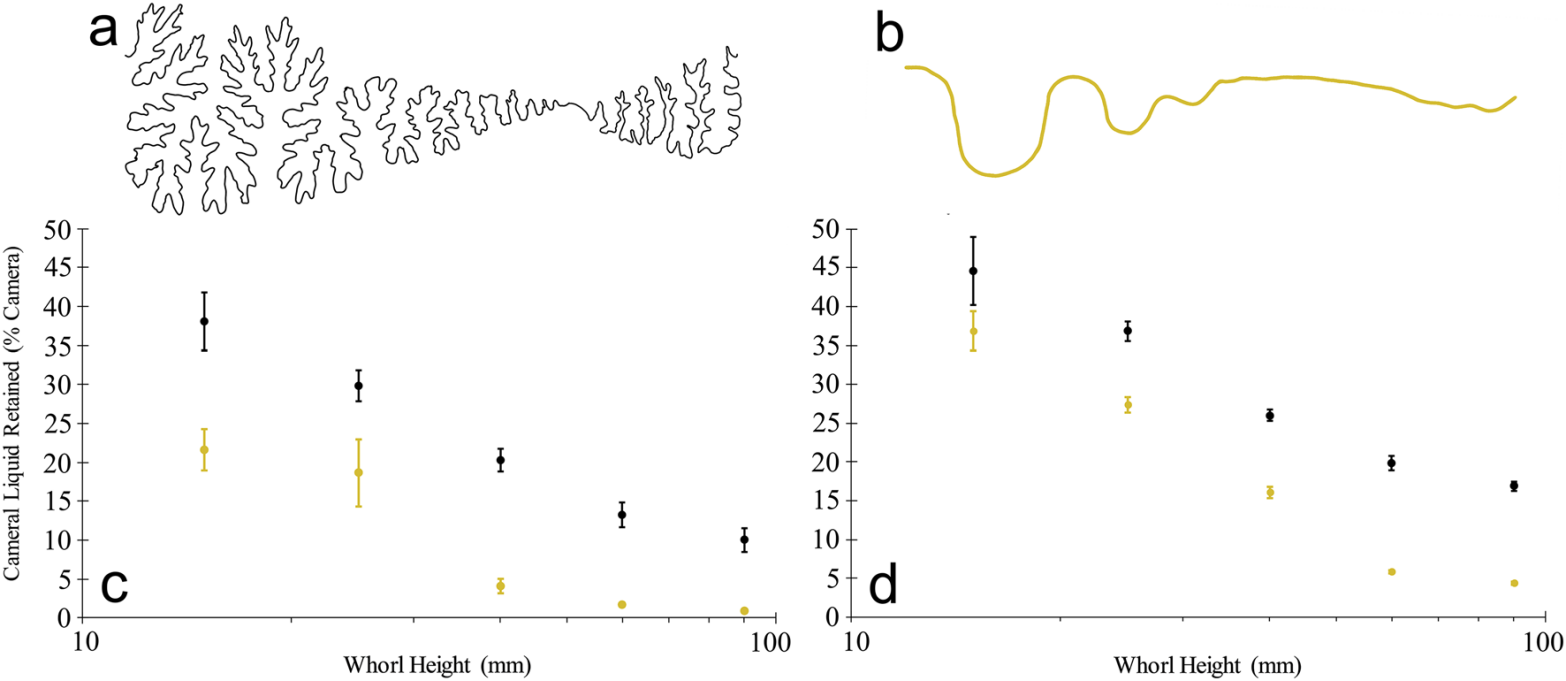
Liquid retention within the 3D printed chamber models of *Damesites* sp. (derived from the CT scan data of Inoue and Kondo^63^). (**a**) Suture pattern (left hemisuture shown) recorded from *Damesites* sp. (**b**) Suture pattern from (**a**) after smoothing to reveal only first order folds. (**c**) Cameral liquid retention (average percent camera at various scales) in the complex and simple *Damesites* models without hydrophilic coating. (**d**) Cameral liquid retention in both models with hydrophilic coating. Whorl heights are dorsoventral lengths measured in the center of the camerae. The black and gold datapoints correspond to models with complex and simple sutures, respectively. Error bars represent one standard deviation (n = 10).

**Figure 5:**
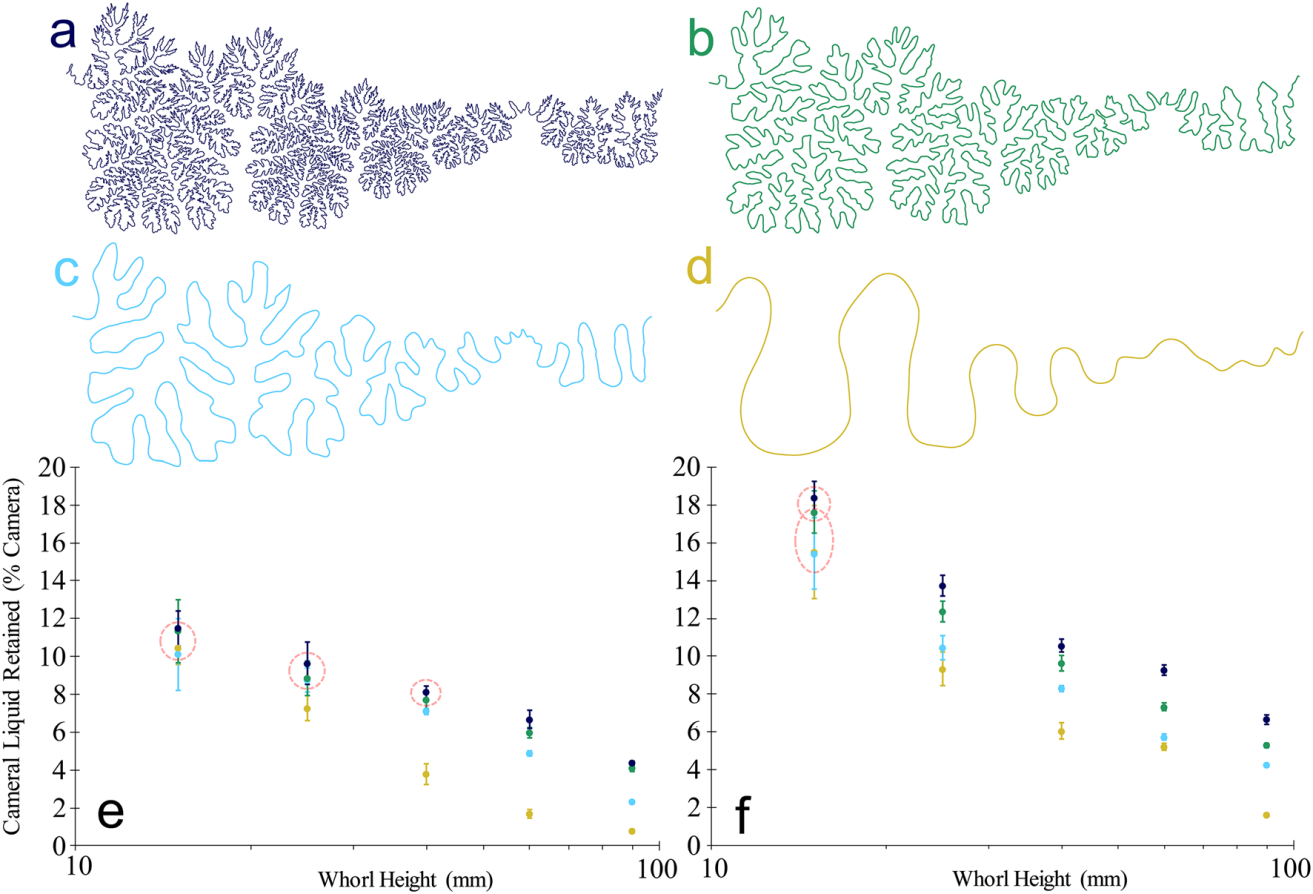
Liquid retention within the 3D printed chamber models of *Menuites oralensis*. The original suture pattern (**a**) was smoothed to successively remove higher order folds (**b**–**d**). Only the left hemisutures of the septa are shown. (**e**) Cameral liquid retention (average percent camera at various scales) in the *Menuites* models with no internal coating. (**f**) Cameral liquid retention in the *Menuites* models with hydrophilic coating. Whorl heights are dorsoventral lengths measured in the center of the camerae. All data points are color-coded according to the sutures in parts (**a**–**d**). Error bars represent one standard deviation (n = 10). Red dashed circles highlight data pairs that cannot be statistically distinguished with a Games-Howell post hoc test (p > 0.05).

The PLA (polylactic acid) plastic models without coating, retain liquid in the septal margins similar to the cylindrical septa experiments. It is difficult to compare liquid retention between species of varying whorl section and septal spacing because their relative chamber volumes differ. For example, the *Menuites* chambers retained less liquid per cameral volume (1/3 to 1/2 depending on the model size and coating) than the *Damesites* chambers even though their margins are much more complex. This is almost entirely due to the lateral inflation of the *Menuites* whorl section. Therefore, changing the complexity of the suture line while holding the other geometric properties of the shell constant is ideal to investigate the relationship between liquid retention and septal complexity. As the chambers increase in size, less liquid is retained in the camerae because the relative size of the water droplets (capillary length) decreases with respect to the size of the septal recesses (Figs. 4 and 5). In living ectocochleates, this capillary length can change depending on the dissolved contents and temperature of cameral liquid. While these properties can adjust the values of liquid retained in living cephalopods, the same relative trend between septal complexity, scale, and liquid retention in the current models are expected to hold true. Like the cylindrical septa models, more liquid is retained in septa with more complex sutures (Figs. 4 and 5). This relationship is generally apparent at all scales examined, although there is more variability in the liquid retained between trials at lower scales, some of which cannot be statistically distinguished (red dashed circles on Fig. 5). This is likely due to the larger influence of surface tension relative to gravity at these scales, and the first-order contours governing the retention of liquid.

Species within the genus *Damesites* are generally small, with terminal septum whorl heights of around 3 cm. Hence, the experiments on larger camerae are hypothetical, and demonstrate cameral liquid retention in larger ammonoids with similar suture patterns. *Menuites*, however, can reach very large sizes, with terminal whorl heights larger than the largest camerae experimented upon in this study (> 9 cm). Therefore, the minor differences between the realistic *Menuites* suture and the suture smoothed over one iteration (Fig. 5B) may be more significant at even larger scales.

The models coated in micro-dispersed oxidized cellulose retain liquid along the entire internal interface of the chambers in addition to the septal margins. By visual inspection, the spray coating was well-distributed along the inside of the camerae. However, if this spray failed to reach the finer scale recesses of the septa, these areas would still be submerged due to the surface tension created by septal contours. In addition to retaining more liquid, these models display the same trends as the uncoated models; (1) a decrease in cameral liquid retention capacity as scale increases, and (2) more complex septa have a greater retention capacity, particularly at larger scales.

A larger capacity for cameral liquid retention in each of these experiments demonstrate an increase in total surface tension potential along septa at a particular scale. These volumes of cameral liquid suggest that septal complexity influences the buoyancy (mass balance) and liquid transport within the camerae.

## Discussion

Suture complexity is a first-order control on passive fluid retention in ammonoid shells. The convoluted shape of the margins, rather than overall surface area, drives the main threshold of this critical biotic feature: the ammonitic sutures, frilled at multiple scales, have higher surface tension potential than the simpler, less frilled forms. Below we discuss possible functional interpretations involving buoyancy management for the most elaborate and ubiquitous shelled cephalopods in history.

Chambers with ammonitic sutures are able to store more water along their fluted flanks, while exploiting low-angle contacts to retain water droplets through simple surface tension. Adding a hydrophilic coating magnifies the effect, and more-closely mimics the capillary conditions within the shells of living cephalopods^25,26^ (Supplementary Fig. 2). These experiments are not designed to measure the exact fluid retained during life in these extinct animals; rather, they allow relative measures of the impact of sutural complexity on liquid retention potential while holding all other morphological features constant (e.g., whorl section shape and septal spacing). Furthermore, they do not suggest that liquid retained in this manner is incapable of being modified or reduced. Osmotic pumping would likely be able to transport liquid against the minor surface tension pressures created by the low-angle septal shapes^21–23,26^. Though, it is unknown how these liquid transport rates would change while emptying chambers bound by complex septa or how these shapes impose pumping limits in nearly emptied chambers. Chambers with non-ammonitic sutures retain less liquid across the board, and the amount of fluid retained is not directly related to the chamber surface area (Fig. 3). Therefore, we restrict our interpretations to ectocochleates specifically with ammonitic sutures. These results refine and support longstanding hypotheses for physiological functions of complex septa, particularly involving improved cameral liquid retention for buoyancy modification^24,25,28,47,54^. Moreover, these experiments demonstrate that the relationship between liquid retention potential and suture complexity is a threshold response, requiring the fractal-like folding of ammonitic septal margins to optimally hold cameral liquid.

The many recesses created by ammonitic frilling of the septal margins allow liquid retention on both sides of each dividing wall, an architectural leap nearly doubling their capacity for passive liquid storage compared to nautilus septa (confined in shells of the same shape and size; Fig. 3). The practical consequences of this feature are easily extrapolated from observations of extant *Nautilus*. Live *Nautilus* retains a small amount of fluid in its shell, primarily along the low angle margins of the convex, adapical sides of each septum^21,26^. The pellicle of extant nautilids thickens at the margins, and after a chamber is effectively emptied, residual liquid remains in this saturated membrane, forming a gel-like mixture of conchiolin, water, and other organic and inorganic components^26,64,65^. The models lined with a hydrophilic coating demonstrate a similar scenario, although the exact nature of this membrane in living ammonoids is largely unknown. If ammonoid membranes were sufficiently thickened at the margins during life, this gel-like mixture may have occupied the entirety of each finer-scale recess for ammonitic septa. Such reserves could have been ideal to store more liquid and adjust its total volume by saturation and desaturation of the membrane.

### Scaling relationships

**Figure 6:**
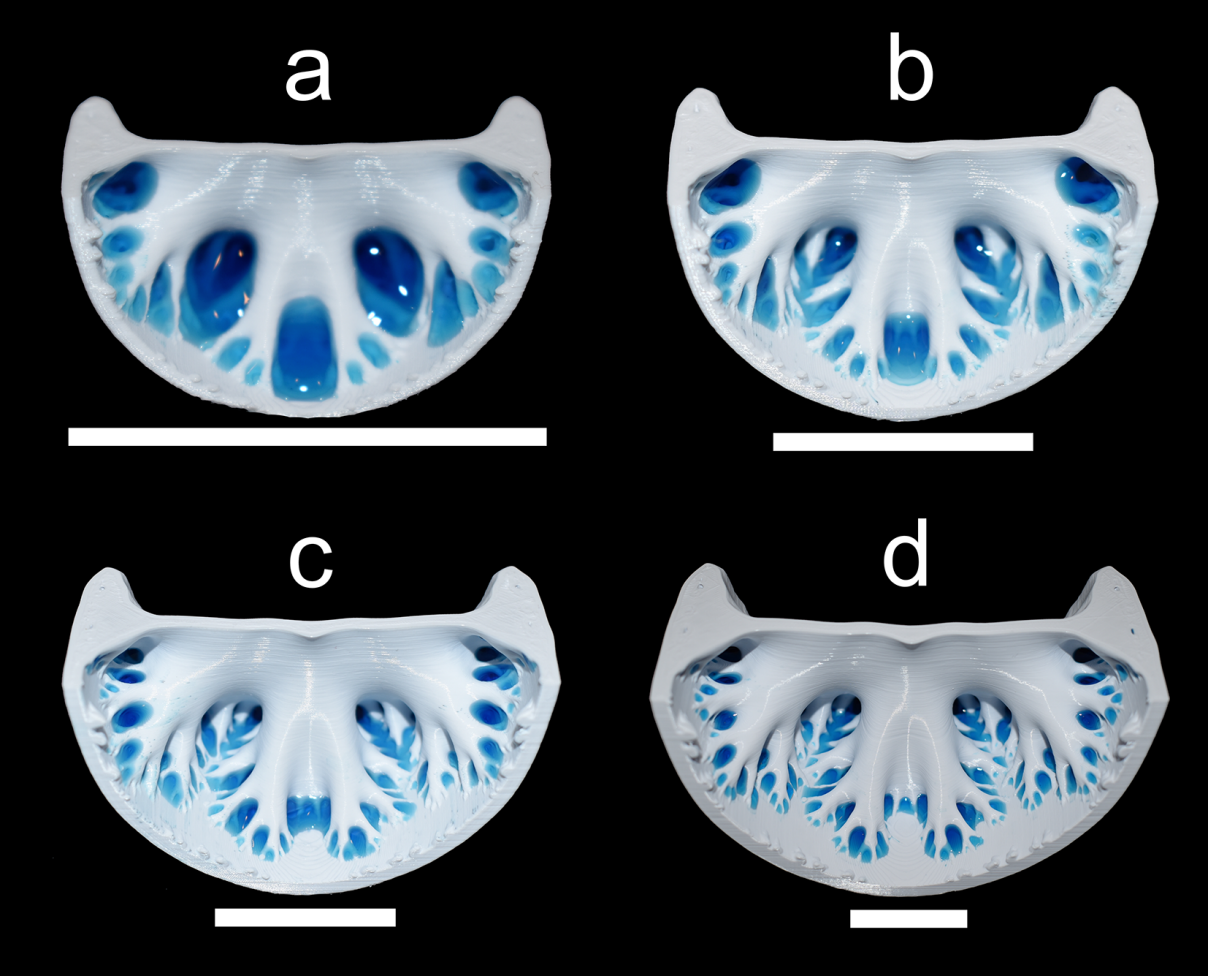
Demonstration of liquid retention along the septal margins of the *Menuites oralensis* chamber models (adapical view). These trimmed models correspond to chambers with whorl heights of 25, 40, 60 and 90 mm (**a**–**d**, respectively). All scale bars = 3 cm. Water is dyed blue for visibility.

The fractal-like self-similarity of ammonitic sutures has direct control on the buoyancy of living ammonoids by influencing liquid retention capacity at multiple scales. In small chambers, the lower-order folds hold “droplets” of water via surface tension and adhesion, which are larger relative to the size of the septum^47^. The higher-order elements in these small chambers are entirely submerged and effectively moot, which explains why complexity does not predict liquid retention in small models (15–25 mm whorl height). As chamber size increases, however, the liquid occupies higher-order folds until only the finest elements carry liquid (Fig. 6). Large chambers without ammonitic sutural complexity retain far less liquid through passive surface tension, creating the threshold response illustrated in our experiments (Figs. 4 and 5). This scaling behavior may explain why sutural complexity is greater for ammonoids with larger sizes^50,55^.

Sutural complexity makes little difference on liquid retention within small camerae. The larger percentages of liquid retained in the chamber models (Figs. 4 and 5) reflect that smaller camerae have a larger capacity for liquid retention simply because the capillary length of the liquid is larger relative to the size of the chamber. Furthermore, it is likely that such high volumes of liquid would need to be managed to avoid strong negative buoyancy^66^. As the living animal grows, the camerae increase in size and the relative capillary length of the cameral liquid decreases. Complex septa compensate for these scaling differences at these larger scales because they retain a larger capacity for liquid storage compared similarly sized camerae with simple septa. The fractal-like branching of the septa (many more folds superimposed on each order of folds) reflects multifunctionality for liquid retention at several scales by retaining liquid in progressively finer septal recesses as chamber size increases (Fig. 6).

### Ecological implications

Passive cameral liquid retention potentially offers advantages for ammonoids with ammonitic sutures. One interpretation is that storage of cameral liquid must add ballast mass to reduce positive buoyancy. These functions are used today in the external shell of extant *Nautilus*, and in the internal segmented shell of extant cuttlefish (the closer relatives to ammonoids^67^). While cuttlefish can use cameral liquid to modify their orientation^68^, it is unknown if the liquid retained in complex ammonoid shells could substantially alter their mass distributions. Estimated liquid volumes required to alter buoyancy in ammonoids vary widely with many features: shell shape; soft-body extent; ontogeny; shell thickness; etc. Live *Nautilus* vary from up to 32% overall fluid retention in juveniles, to about 12% or less in adults^19,21^. Estimates for ammonoids range from nearly empty to 40%^56–60^. Alternatively, positive buoyancy could have been offset by simply increasing the relative size of the soft body instead of retaining more liquid. Perhaps, retaining liquid in this manner was advantageous for improving liquid transport or the modification of these liquid volumes. The range of retention volumes shown in our experiments suggests there should be some critical, but perhaps complex, relationship to primary hydrostatics for the full spectrum of ammonitic sutural complexity.

A larger capacity for cameral liquid storage could have allowed ammonitic septa to house liquid reserves for greater ranges of buoyancy adjustment. However, it is unknown whether ammonoids could have utilized positive or negative buoyancy differences for vertical migration in the water column. Liquid transport is a slow process for extant nautilids, which primarily use active locomotion for vertical movement, even over large distances^69,70^. As postulated by Daniel et al.^47^, the frilled margins of ammonitic septa would have created larger surface tension pressures, possibly increasing the rate of chamber refilling. The experiments in the current study suggest that more septal recesses at each appropriate scale could increase the accommodation space for cameral liquid refilling due to surface tension. Larger volumes of water retained against gravity in these experiments demonstrate that increasing the number of folds with low radii of curvature, raises the potential surface tension pressures along the periphery of each septum. When osmotic pumping is reduced by the living animal, these numerous high surface tension zones could have improved liquid refilling^47^. A larger capacity for buoyancy reduction could have facilitated downward movement in the water column, or acted as a compensation mechanism for sudden increases in buoyancy, such as shell breakage near the aperture^47,65,71^. This interpretation is reinforced by the higher tolerance for shell loss in ammonoids compared to nautiloids^71^. In lytoceratids, the dorsal portion of the septum reaches apically and frills, covering a portion of the previous septum. This increase in septal surface and the additional frilled margins of these unique septal lobes warrant investigation of their possible roles in buoyancy modification^72^.

Retaining more liquid may have also allowed countermeasures against accumulated mass, resulting in negative buoyancy. Such scenarios involve removing surplus cameral liquid to compensate for the mass of epibionts^73^, and perhaps the mass incorporated by feeding or reproduction^65^. In contrast, a surplus or deficiency in buoyancy can be settled by adjusting the volume of the soft body during growth^74^, or simply by adding another chamber. Perhaps ammonitic septa facilitated quicker buoyancy modification compared to these other tactics. In terms of cameral liquid removal, larger septal surfaces have been interpreted to increase liquid transport to the siphuncle^52^. However, the surface area increase by ammonitic septa is not optimal and can be the result of adding more primary septal folds (e.g., *Schistoceras*; Fig. 3) or reaching further into the longitudinal directions. While these short-term compensation mechanisms for increased mass are possible, they do not suffice for sole explanations of increased sutural complexity through time. Each of these outlined interpretations, however, provide testable hypotheses regarding buoyancy modification and cameral liquid transport in species with ammonitic septa. The methods of the current study could be adapted to further explore rates of cameral liquid transport (involved in shell growth or buoyancy compensation).

Finally, each advantage outlined above interacts with the overall shape and size of ammonoid shells, giving critical ecological context to vital events in evolution and extinction. Ammonoids are key tracers of biotic crises, and shell shape proves selective in many extinction and diversification events^75–77^. Changes in ammonoid conch morphology may not only reflect selection for favorable hydrodynamic properties^78^, but also different strategies for utilizing their shells as buoyancy apparatuses. For example, laterally-compressed shells would have incurred lower hydrodynamic drag^79^, while simultaneously increasing the liquid retention capacity per chamber. This larger capacity would be the result of decreasing empty space in the chambers while relatively increasing the internal areas of the shell wall; thus, improving surface tension potential along the crenulated septal margins. In contrast, inflated and evolute shells would require increased sutural complexity to increase surface tension potential. Similarly, more densely packed septa would decrease this empty space in each camera. Conch morphology also influences septal morphology, in addition to hydrostatics. The relationship between suture complexity and conch shape has been well documented, often involving covariation between shell compression^80–82^. The interrelation of conch morphology, septal morphology, and hydrostatics may help elucidate the mechanisms driving septal complexity and its iterative recurrence throughout ammonoid evolution. Our results suggest that extinction events involving ammonoids with ammonitic sutures may have been paired with shifts in buoyancy management for the surviving cephalopods. These relationships should be investigated in future studies concerning both selection and the influence of various aspects of conch morphology.

The iterative trend of increasing sutural complexity is enigmatic and is not likely to be explained for all ammonoids by a single selection mechanism. After all, there are many examples of homeomorphic taxa exhibiting morphologically similar sutures in clades that are not closely related. These taxa suggest an adaptive value for each suture type is possible if they are actually the result of functional convergence and not governed by fabricational noise^34^ or indirect changes in conch morphology (e.g., micromorphy, siphuncle position). The selective value of different suture types can also depend on other indirect factors. While ammonoids with ammonitic sutures flourished after the end-Triassic mass extinction, they met their demise at the Cretaceous-Paleogene mass extinction, after reaching their height of their sutural complexity in the Jurassic and Cretaceous^6^ (Fig. 1F). Ironically, only the simply-sutured nautiloids survived into the Cenozoic, suggesting that the higher fecundity, smaller embryos (with less reserves), planktic hatchlings, and potential metabolic differences of the ammonoids outweighed differences in conch architecture and septal complexity as critical traits during this event^32,33,83,84^.

Overall, we demonstrate that suture complexity is a first-order control on mass balance in cephalopods and presents direct ecological consequences for the famed fractal-like morphology of ammonitic septa. Our results support previous studies that suggest ammonoids may have had more active buoyancy regulation compared to extant nautilids^47,52,71^. These experiments offer a host of easily-tested hypotheses for the interaction of architectural complexity, functional morphology, and evolutionary success in this diverse clade. Ammonoids have long presented a prime example of an increase in biological complexity through time. Our experiments show fundamental ecological consequences for that complexity, and its critical role in ammonoid evolution.

## Materials and methods

Two types of models were virtually constructed and 3D-printed for experimentation: (1) theoretical, cylindrical shells, containing a single septum (Fig. 2C), and (2) entire camerae, printed at various sizes (Fig. 2F). The capillary properties of the models were also modified for comparison, and to create a lining similar to the cameral membranes of extant ectocochleate cephalopods^26^.

### Cylindrical shell models

Each of the cylindrical shell models were created in a similar method to those used in the compression experiments by Johnson et al.^46^. Seven suture patterns were chosen to capture a wide range of sutural complexity: *Orthoceras regulare* (nautiloid; Orthocerida), *Manticoceras* sp. (simple goniatitic; Agoniatitida), *Schistoceras missouriense* (complex goniatitic; Goniatitida), *Xenodiscus* (*Xenaspis*) *carbonarius* (simple ceratitic; Ceratitida), *Otoceras woodwardi* (complex ceratitic; Ceratitida), *Baculites compressus* (simple ammonitic; Ammonitida), and *Diplomoceras notabile* (complex ammonitic; Ammonitida). A simple and complex suture (in relative terms only) was chosen for each of the three ammonoid suture categories due to the extreme morphological disparity within this group. The geometries of the shells containing each of the septa were held constant. These shells were created in Blender (Blender Foundation) as cylinders 60 mm in diameter, 45 mm in length (slightly larger than the longitudinal length of the largest suture; *Diplomoceras*), and extruded at 0.6 mm (the minimum wall thickness suitable for 3D printing). Septa were constructed by importing the 2D suture pattern and using the curve modifier in Blender to wrap it around the internal face of the cylindrical whorl section (Fig. 2A). This suture was then repaired to create a closed loop. The nautiloid septum was created by making a hemisphere, matching the radius of curvature of *Orthoceras regulare* septa (Teichert et al.^85^; pg. 225, fig. 153). Ammonoid septa were extruded inwards to the centroid of the shell. The crude septa constructed by this method were then refined (improving resolution with more faces and vertices), and smoothed to approximate minimum curvature surfaces (Fig. 2A). The finished septum was then extruded to 0.6 mm then unified to the external shell model using the Boolean modifier in Netfabb (Autodesk Inc.), creating the final virtual model (Fig. 2B). The final virtual models were 3D-printed in ABS-P430 filament with a Stratasys Dimension Elite 3D printer. Support structures were printed with the secondary extruder in Stratasys SUP706 filament and dissolved in an ultrasonic bath of NaOH solution.

Each 3D-printed model was weighed dry, dunked in tap water, then weighed again to compute the mass of water retained by the septal margins. Before weighing, the wet models were rotated in many different orientations for approximately 30 s on each side. Doing so released liquid from recesses that was only held by gravity and not by surface tension. Furthermore, the external portions of the cylinders were dried before weighing. While liquid adhered to the ABS plastic alone, the capillary properties of these surfaces were adjusted to mimic the pellicle lining of living ectocochleate cephalopods. A hydrophilic surface was created by spraying micro-dispersed oxidized cellulose on the models. For comparison, a third set of models were treated with a hydrophobic silicone spray. Each of the coated models were allowed to dry before measuring their new dry masses. Averages of retained liquid masses were computed for 10 trials on each model. Surface areas were computed in MeshLab^86^ in order to compare with the liquid retained at septal margins and throughout the entire models with hydrophilic coatings.

### Chamber models

While the cylindrical shell models are useful to understand relative differences in liquid retention between different septal margins, they are not without limitations. Most importantly, the cephalopods exhibiting these different suture patterns vary in whorl section shape, septal spacing, size, and other characters. Scale is an important property in many biological principles, and the models mentioned above cannot be printed much smaller than 60 mm whorl height due to the limitations of wall thickness (~ 0.6 mm). To overcome this, individual chambers (camerae; Fig. 2D) bound by two septa were constructed for two species, *Damesites* sp. and *Menuites oralensis* (the latter considerably more complex). The *Damesites* camera was extracted from the CT (computed tomography) scan from Inoue and Kondo^63^. The *Menuites* camera was constructed using the methods of Peterman et al.^59,60^. A specimen of *Menuites oralensis* was prepared to expose the entire suture pattern (including the internal portion). Photogrammetry was used to model the whorl section in 3DF Zephyr (3DFlow). The method used to create the cylindrical septa was used to construct the *Menuites* septum via the whorl section from the photogrammetry model instead of a cylinder. The septum was replicated, and its copy positioned so that each of the distal lobules and folioles were tangential. Each virtual camera was subtracted from arbitrary volumes that bound them (~ 3 to 5 mm larger) using the Boolean modifier in Netfabb (Autodesk Inc.) (Fig. 2E). Liquid drainage tubes were also subtracted from the model with the same process. The camerae were scaled to whorl heights (dorsoventral lengths in the middle of each chamber) of 15, 25, 40, 60, and 90 mm in order to compare how liquid retention changes with scale. The final chamber models were 3D-printed with an Ultimaker S5 3D Printer in PLA filament (Fig. 2F). The *Damesites* suture was smoothed to yield only the first order folds and chamber models were printed following the procedure above. The *Menuites* suture, being much more complicated, was smoothed over three iterations, producing four varieties of models with varying sutural complexity. The smoothing process was performed on the septa with a Laplacian smoothing algorithm in Blender. The smooth tool in Meshmixer 3.3 (Autodesk Inc.) was used to remove remaining higher-order folds that were differentially preserved along the more complex regions of the septa (lateral and ventral areas).

All of the chamber models were re-printed in two separate halves in order to apply the micro-dispersed oxidized cellulose. Painter’s tape was used to prevent the spray from contacting the halves of plastic, which were later joined with cyanoacrylate glue. A syringe was used to deliver water to the chambers. Each 3D-printed model was rotated at varying orientations and allowed to drain through each of the three drainage tubes (for ~ 30 s on the ventral, adoral, and adapical sides). A syringe was used to assist draining in the smaller models (15 and 25 mm whorl heights) since surface tension would prevent drainage through their smaller tubes. Like the cylindrical models, the masses of the dry models were subtracted from the masses of the water saturated models to measure the mass of liquid retained by surface tension. Measurements were repeated 10 times for each model to compute average masses of liquid retained. In order to compare different scales to each other, the percentage of liquid retained in each chamber was computed by dividing the mass of the water retained by its density, then dividing by the cameral volume of that model. Volumes were computed from the virtual chamber models in MeshLab 1.3.3^86^.

A one-way analysis of variance (ANOVA) with a Games-Howell post hoc test was performed to determine if the differences in mean liquid retention values between models of neighboring sutural complexities were significant (Supplementary Tables 1–10). This method was chosen because it works well with low sample sizes and does not require the assumption of equal variance between samples. Pairs with p-values less than 0.05 were deemed indistinguishable.

## Supplementary Information

Supplementary Information.

## Data Availability

The digital models are available in an online repository (10.5281/zenodo.4308813).
