# Supplementary material for: Buoyancy control in ammonoid cephalopods refined by complex internal shell architecture
Source: Sci Rep. 2021 Apr 13;11:8055. doi: 10.1038/s41598-021-87379-5 (PMC8044186; doi:10.1038/s41598-021-87379-5)
Supplement: Supplementary file 1 — Supplementary Information. [file 41598_2021_87379_MOESM1_ESM.docx]

**Supplementary Information for**

Buoyancy control in ammonoid cephalopods refined by complex internal shell architecture

David J. Peterman^1*^, Kathleen A. Ritterbush^1^, Charles N. Ciampaglio^2^, Erynn H. Johnson^3^, Shinya Inoue^4^, Tomoyuki Mikami^5^, and Thomas J. Linn^6^

^1^Department of Geology and Geophysics, University of Utah, UT 84112. ^2^Department of Science, Mathematics, and Engineering, Wright State University (Lake Campus), Celina, OH 45822. ^3^Paleontological Research Institution, Ithaca, NY 14850. ^4^Hokkaido University Shuma-no-kai, Hokkaido 060-0817, Japan. ^5^Department of Biological Sciences, University of Tokyo, Tokyo 113-0032, Japan. ^6^Division of Paleontology, Frontier Gateway Museum, Glendive, MT 59330

*David J. Peterman – Corresponding Author (David.Peterman@utah.edu).

**This PDF file includes:**

Supplementary text

Supplementary Figures 1 and 2

Supplementary Tables 1 to 12

Legends for Dataset 1

SI References

**Other supplementary materials for this manuscript include the following:**

Dataset 1

Supplementary Information Text

**Overview.** The supplementary tables (Tables 1-10) contain the results of the cameral liquid retention experiments for the cylindrical septa models and chamber models. These tables include post hoc tests to determine if the differences between means are significant. An additional experiment using a chamber from a modern *Nautilus* shell is described below. The artificial hydrophilic membrane used in all experiments was compared with the *Nautilus* chamber, and demonstrated with an additional supplementary figure. The files used to 3D-print the cameral liquid retention models are deposited in a separate repository (described in the Dataset 1 section below).

Supplementary materials and methods

***Nautilus pompilius camera experiments*.** To investigate the capillary similarities between the artificial hydrophilic coating (micro-dispersed oxidized cellulose) and the pellicle membrane of living ectocochleates (i.e., nautilids), a single chamber from a modern *Nautilus pompilius* shell was examined. This chamber was extracted from the shell by cutting it along its diameter with a wet saw, then using a diamond-plated, Dremel cutting tool to remove the extraneous shell. The percent liquid retained in this single chamber (Fig. 1A, B) was measured under three conditions: 1) with the original, desiccated pellicle membrane, 2) after scraping most of the pellicle membrane away, and 3) with micro-dispersed oxidized cellulose sprayed into the chamber. The latter two experiments were conducted on the chamber after cutting it in half (Fig. 1C). The shell was weighed dry for each condition, then saturated with a syringe, and weighed wet. The difference in these two masses was computed to find the mass of liquid retained. The percentage of liquid retained was found by dividing the liquid mass by its density, then dividing by the empty chamber volume.

**Hydrophilic coating on 3D-printed models.** The hydrophilic coating applied to all 3D-printed models is a wettable, capillary surface, meant to mimic the pellicle membrane of living ectocochleate cephalopods. The capillary properties of this artificial coating (micro-dispersed oxidized cellulose) were visually demonstrated by dropping water (dyed blue) in the center of one of the cylindrical septa models (*Manticoceras sp.*).

Supplementary results and discussion

**Cameral liquid retention in a modern *Nautilus* chamber.** Adult *Nautilus* retain about 12% cameral liquid within their phragmocones^1,2^. If each chamber retained this same proportion of cameral liquid, their sum would equal 12% of the entire phragmocone as well. Therefore, the liquid retention experiment on the modern *Nautilus pompilius* chamber with micro-dispersed oxidized cellulose demonstrates that this artificial hydrophilic coating may have similar hydrophilic properties to the syn vivo pellicle membrane of extant ectocochleates. With the artificial coating, the average percentage of liquid retained in this chamber is around 11.9% (Table 11). However, such a direct comparison has its caveats. It should be noted that the chamber experimented upon is the last one in the adult shell (i.e., experiences septal crowding, like many adult ectocochleates^3^). Therefore, the overall chamber volume is lower by some amount, potentially increasing the percentage of liquid retained compared to younger camerae of similar size. Furthermore, the camerae experiments demonstrate that more liquid is retained at smaller scales, so the percentage of liquid retained in the entire phragmocone may be different with the artificial coating. In living nautilids, cameral liquid measurements depend on how much liquid has yet to be drained in the most recent chamber. For nautilids with flooded terminal camerae, the measurements of liquid retention by surface tension cannot be directly compared. While these relationships between living nautilids and the single chamber experiment are difficult to disentangle, the properties of the artificial coating yield realistic values of a hydrophilic membrane that satisfies the purposes of this study—allowing relative measures of liquid retention to be investigated.

The original, desiccated pellicle membrane in the *Nautilus* chamber does not significantly influence liquid retention. Measured liquid retained during this condition is not distinguishable from measurements of the same chamber with the pellicle scraped away (Table 12). This indifference suggests that the pellicle of a long-dead *Nautilus* no longer behaves like that of a living one.

**Demonstration of the hydrophilic membrane.** The micro-dispersed oxidized cellulose acts as a wettable film that draws in water and retains it against gravity. After applying liquid to the center of the cylindrical model, it slowly absorbed into the coating (Fig. 2), with gravity simultaneously dispersing the liquid to the low spots. This simple demonstration verifies that liquid is retained along the septal surface with this coating, in addition to enhanced retention in the septal recesses.


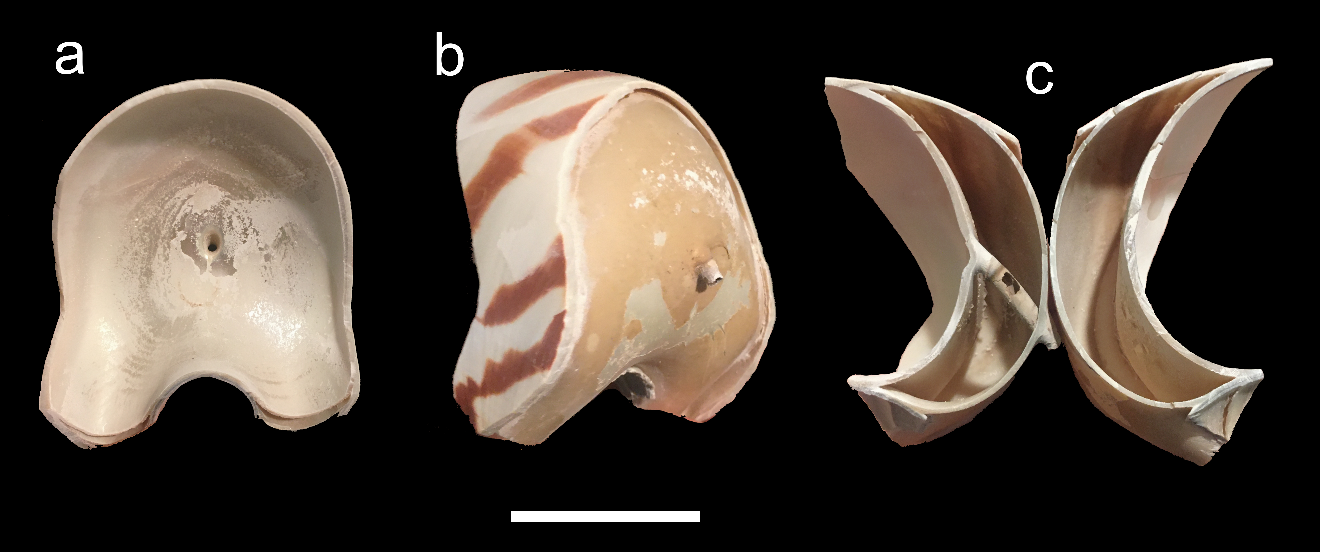


Fig. 1. Single chamber of a modern *Nautilus pompilius* shell (terminal camera at whorl height of 62 mm). A) View of adapertural side. B) oblique view showing adapical side. C) Half cut chamber allowing micro-dispersed oxidized cellulose coating to be added. Scale bar = 3 cm.


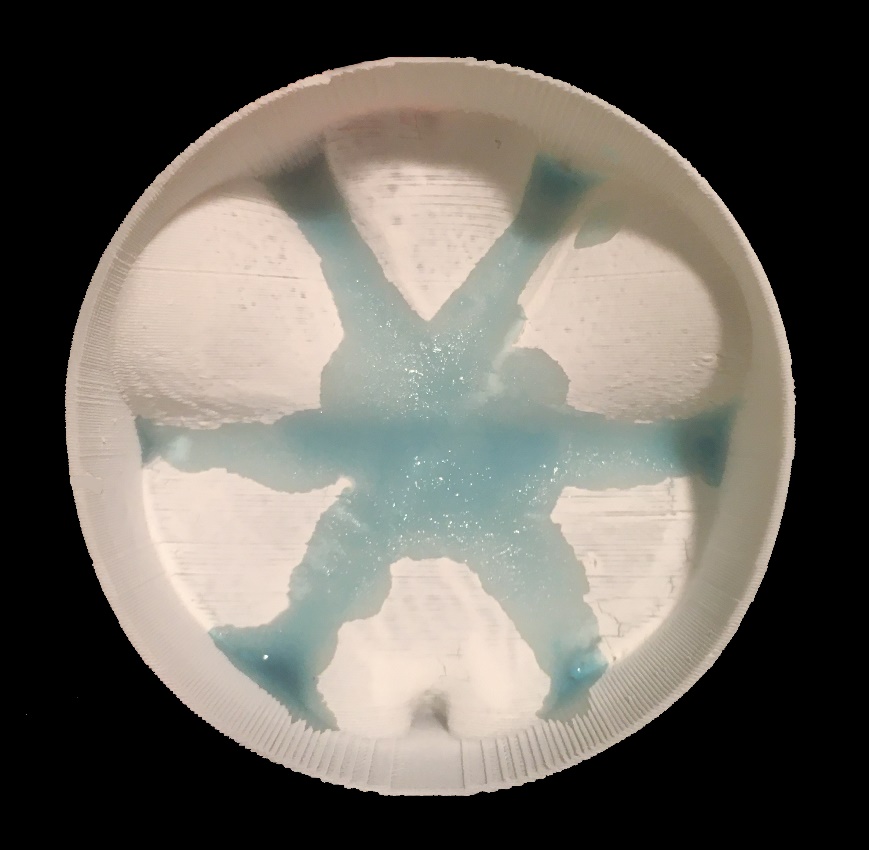


Fig. 2. Cylindrical septum model with a *Manticoceras sp.* suture (60mm in diameter). Water was dyed blue and applied to the model to demonstrate the wettable, capillary properties of the artificial coating (micro-dispersed oxidized cellulose).

Table 1. Mean liquid retained (in grams) in the cylindrical septa experiments and the standard deviation (std. dev.) between trails (number of measurements (n) for each model is 10).

|  | No coating | | Hydrophilic coating | | Hydrophobic coating | | Septal Surface Area (cm^2^) |
| --- | --- | --- | --- | --- | --- | --- | --- |
| group | mean | std. dev. | mean | std. dev. | mean | std. dev. |  |
| *Orthoceras* | 0.5608 | 0.0951 | 3.0345 | 0.0254 | 0.2527 | 0.0286 | 34.19 |
| *Manticoceras* | 0.5972 | 0.0880 | 3.6219 | 0.0254 | 0.2394 | 0.0184 | 35.47 |
| *Schistoceras* | 1.1035 | 0.1313 | 5.2692 | 0.0386 | 0.5827 | 0.0385 | 46.07 |
| *Xenaspis* | 0.7708 | 0.0613 | 3.5997 | 0.0452 | 0.2743 | 0.0311 | 38.48 |
| *Otoceras* | 1.3419 | 0.3132 | 5.0404 | 0.3328 | 0.5076 | 0.0364 | 34.40 |
| *Baculites* | 1.8307 | 0.1023 | 7.6839 | 0.0381 | 0.8622 | 0.1037 | 42.76 |
| *Diplomoceras* | 4.2288 | 0.3385 | 9.7928 | 0.0919 | 1.6916 | 0.0852 | 91.12 |

Table 2. Games-Howell post hoc test for mean differences in liquid retained (in grams) between each cylindrical septum model without coating. Lower and upper 95% confidence intervals and p-values are reported at a confidence level (α) of 0.05. The number of measurements (n) for each model is 10. Highlighted p-values are greater than 0.05 and are deemed statistically indistinguishable.

| Q TEST | | No coating | | | | |
| --- | --- | --- | --- | --- | --- | --- |
| group 1 | group 2 | mean | std err | lower | upper | p-value |
| *Orthoceras* | *Manticoceras* | 0.0364 | 0.0290 | -0.0991 | 0.1718 | 0.970 |
| *Orthoceras* | *Schistoceras* | 0.5427 | 0.0362 | 0.3714 | 0.7140 | 1.722E-07 |
| *Orthoceras* | *Xenaspis* | 0.2100 | 0.0253 | 0.0894 | 0.3305 | 4.444E-04 |
| *Orthoceras* | *Otoceras* | 0.7811 | 0.0732 | 0.4108 | 1.1515 | 1.949E-04 |
| *Orthoceras* | *Baculites* | 1.2699 | 0.0312 | 1.1239 | 1.4159 | 2.609E-14 |
| *Orthoceras* | *Diplomoceras* | 3.6681 | 0.0786 | 3.2685 | 4.0676 | 5.035E-11 |
| *Manticoceras* | *Schistoceras* | 0.5064 | 0.0353 | 0.3384 | 0.6743 | 4.749E-07 |
| *Manticoceras* | *Xenaspis* | 0.1736 | 0.0240 | 0.0600 | 0.2873 | 1.581E-03 |
| *Manticoceras* | *Otoceras* | 0.7448 | 0.0728 | 0.3750 | 1.1145 | 3.138E-04 |
| *Manticoceras* | *Baculites* | 1.2335 | 0.0302 | 1.0922 | 1.3749 | 2.975E-14 |
| *Manticoceras* | *Diplomoceras* | 3.6317 | 0.0782 | 3.2327 | 4.0307 | 8.127E-11 |
| *Schistoceras* | *Xenaspis* | 0.3328 | 0.0324 | 0.1740 | 0.4915 | 1.099E-04 |
| *Schistoceras* | *Otoceras* | 0.2384 | 0.0759 | -0.1372 | 0.6139 | 0.352 |
| *Schistoceras* | *Baculites* | 0.7272 | 0.0372 | 0.5520 | 0.9023 | 2.115E-09 |
| *Schistoceras* | *Diplomoceras* | 3.1253 | 0.0812 | 2.7214 | 3.5293 | 1.112E-10 |
| *Xenaspis* | *Otoceras* | 0.5711 | 0.0714 | 0.2030 | 0.9393 | 2.996E-03 |
| *Xenaspis* | *Baculites* | 1.0599 | 0.0267 | 0.9321 | 1.1878 | 7.283E-13 |
| *Xenaspis* | *Diplomoceras* | 3.4581 | 0.0769 | 3.0604 | 3.8558 | 4.036E-10 |
| *Otoceras* | *Baculites* | 0.4888 | 0.0737 | 0.1177 | 0.8599 | 8.592E-03 |
| *Otoceras* | *Diplomoceras* | 2.8869 | 0.1031 | 2.4047 | 3.3691 | 2.493E-12 |
| *Baculites* | *Diplomoceras* | 2.3982 | 0.0791 | 1.9980 | 2.7983 | 7.084E-09 |

Table 3. Games-Howell post hoc test for mean differences in liquid retained (in grams) between each cylindrical septum model with hydrophilic coating. Lower and upper 95% confidence intervals and p-values are reported at a confidence level (α) of 0.05. The number of measurements (n) for each model is 10. Highlighted p-values are greater than 0.05 and are deemed statistically indistinguishable.

| Q TEST | | Hydrophilic coating | | | | |
| --- | --- | --- | --- | --- | --- | --- |
| group 1 | group 2 | mean | std err | lower | upper | p-value |
| *Orthoceras* | *Manticoceras* | 0.5874 | 0.0080 | 0.5499 | 0.6249 | 1.543E-14 |
| *Orthoceras* | *Schistoceras* | 2.2347 | 0.0103 | 2.1855 | 2.2839 | 1.998E-15 |
| *Orthoceras* | *Xenaspis* | 0.5652 | 0.0116 | 0.5093 | 0.6211 | 4.796E-14 |
| *Orthoceras* | *Otoceras* | 2.0059 | 0.0746 | 1.6156 | 2.3962 | 1.758E-07 |
| *Orthoceras* | *Baculites* | 4.6494 | 0.0102 | 4.6007 | 4.6981 | 1.443E-15 |
| *Orthoceras* | *Diplomoceras* | 6.7583 | 0.0213 | 6.6499 | 6.8667 | -4.317E-12 |
| *Manticoceras* | *Schistoceras* | 1.6473 | 0.0103 | 1.5981 | 1.6965 | 1.221E-15 |
| *Manticoceras* | *Xenaspis* | 0.0222 | 0.0116 | -0.0337 | 0.0781 | 0.816 |
| *Manticoceras* | *Otoceras* | 1.4185 | 0.0746 | 1.0282 | 1.8088 | 3.564E-06 |
| *Manticoceras* | *Baculites* | 4.0620 | 0.0102 | 4.0133 | 4.1107 | -8.882E-16 |
| *Manticoceras* | *Diplomoceras* | 6.1709 | 0.0213 | 6.0625 | 6.2793 | -4.319E-12 |
| *Schistoceras* | *Xenaspis* | 1.6695 | 0.0133 | 1.6072 | 1.7318 | 2.420E-14 |
| *Schistoceras* | *Otoceras* | 0.2288 | 0.0749 | -0.1617 | 0.6193 | 0.395 |
| *Schistoceras* | *Baculites* | 2.4147 | 0.0121 | 2.3580 | 2.4714 | 1.488E-14 |
| *Schistoceras* | *Diplomoceras* | 4.5236 | 0.0223 | 4.4134 | 4.6338 | 9.948E-14 |
| *Xenaspis* | *Otoceras* | 1.4407 | 0.0751 | 1.0501 | 1.8313 | 2.606E-06 |
| *Xenaspis* | *Baculites* | 4.0842 | 0.0132 | 4.0223 | 4.1461 | 2.176E-14 |
| *Xenaspis* | *Diplomoceras* | 6.1931 | 0.0229 | 6.0814 | 6.3048 | 2.588E-13 |
| *Otoceras* | *Baculites* | 2.6435 | 0.0749 | 2.2530 | 3.0340 | 1.191E-08 |
| *Otoceras* | *Diplomoceras* | 4.7524 | 0.0772 | 4.3597 | 5.1451 | 5.347E-12 |
| *Baculites* | *Diplomoceras* | 2.1089 | 0.0222 | 1.9988 | 2.2190 | 1.565E-14 |

Table 4. Games-Howell post hoc test for mean differences in liquid retained (in grams) between each cylindrical septum model with hydrophobic coating. Lower and upper 95% confidence intervals and p-values are reported at a confidence level (α) of 0.05. The number of measurements (n) for each model is 10. Highlighted p-values are greater than 0.05 and are deemed statistically indistinguishable.

| Q TEST | | Hydrophobic coating | | | | |
| --- | --- | --- | --- | --- | --- | --- |
| group 1 | group 2 | mean | std err | lower | upper | p-value |
| *Orthoceras* | *Manticoceras* | 0.0133 | 0.0076 | -0.0230 | 0.0496 | 0.869 |
| *Orthoceras* | *Schistoceras* | 0.3300 | 0.0107 | 0.2794 | 0.3806 | 2.172E-12 |
| *Orthoceras* | *Xenaspis* | 0.0216 | 0.0095 | -0.0226 | 0.0658 | 0.675 |
| *Orthoceras* | *Otoceras* | 0.2549 | 0.0104 | 0.2062 | 0.3036 | 5.202E-11 |
| *Orthoceras* | *Baculites* | 0.6095 | 0.0240 | 0.4872 | 0.7318 | 5.761E-08 |
| *Orthoceras* | *Diplomoceras* | 1.4389 | 0.0201 | 1.3379 | 1.5399 | -2.400E-12 |
| *Manticoceras* | *Schistoceras* | 0.3433 | 0.0095 | 0.2966 | 0.3900 | 4.291E-11 |
| *Manticoceras* | *Xenaspis* | 0.0349 | 0.0081 | -0.0039 | 0.0737 | 0.091 |
| *Manticoceras* | *Otoceras* | 0.2682 | 0.0091 | 0.2238 | 0.3126 | 2.594E-10 |
| *Manticoceras* | *Baculites* | 0.6228 | 0.0235 | 0.5010 | 0.7446 | 1.084E-07 |
| *Manticoceras* | *Diplomoceras* | 1.4522 | 0.0195 | 1.3520 | 1.5524 | 1.312E-11 |
| *Schistoceras* | *Xenaspis* | 0.3084 | 0.0111 | 0.2564 | 0.3604 | 5.575E-12 |
| *Schistoceras* | *Otoceras* | 0.0751 | 0.0119 | 0.0197 | 0.1305 | 4.475E-03 |
| *Schistoceras* | *Baculites* | 0.2795 | 0.0247 | 0.1560 | 0.4030 | 7.681E-05 |
| *Schistoceras* | *Diplomoceras* | 1.1089 | 0.0209 | 1.0062 | 1.2116 | 7.289E-13 |
| *Xenaspis* | *Otoceras* | 0.2333 | 0.0107 | 0.1831 | 0.2835 | 2.274E-10 |
| *Xenaspis* | *Baculites* | 0.5879 | 0.0242 | 0.4654 | 0.7104 | 6.581E-08 |
| *Xenaspis* | *Diplomoceras* | 1.4173 | 0.0203 | 1.3159 | 1.5187 | -1.199E-12 |
| *Otoceras* | *Baculites* | 0.3546 | 0.0246 | 0.2315 | 0.4777 | 7.924E-06 |
| *Otoceras* | *Diplomoceras* | 1.1840 | 0.0207 | 1.0817 | 1.2863 | 9.018E-13 |
| *Baculites* | *Diplomoceras* | 0.8294 | 0.0300 | 0.6886 | 0.9702 | 5.587E-12 |

Table 5. Mean and standard deviation (std. dev.) of the percentage liquid retained in the chamber models of *Damesites*. C = complex suture, S = simple suture. The number of measurements for each model type (n) is 10.

| *Damesites* | No coating | | Hydrophilic coating | |
| --- | --- | --- | --- | --- |
| group (15 mm) | mean | std. dev. | mean | std. dev. |
| C | 38.3193 | 3.34663 | 44.5881 | 4.35091 |
| S | 21.5667 | 2.56997 | 36.8426 | 2.54834 |
| group (25 mm) | mean | std. dev. | mean | std. dev. |
| C | 29.8937 | 1.90723 | 36.8261 | 1.29029 |
| S | 18.7051 | 4.18312 | 27.3811 | 0.95507 |
| group (40 mm) | mean | std. dev. | mean | std. dev. |
| C | 20.3827 | 1.34211 | 26.0116 | 0.74618 |
| S | 3.99855 | 0.88193 | 16.0701 | 0.74292 |
| group (60 mm) | mean | std. dev. | mean | std. dev. |
| C | 13.2069 | 1.50332 | 19.8397 | 0.95703 |
| S | 1.7586 | 0.25428 | 5.83613 | 0.15164 |
| group (90 mm) | mean | std. dev. | mean | std. dev. |
| C | 10.1624 | 1.4855 | 16.8679 | 0.60646 |
| S | 0.85587 | 0.1632 | 4.38832 | 0.17304 |

Table 6. Games-Howell post hoc test for mean differences in liquid retained (percent of camera) between the complex and simple chamber models of *Damesites* (with no coating). Lower and upper 95% confidence intervals and p-values are reported at a confidence level (α) of 0.05. The number of measurements (n) for each model is 10.

| W_h_ (mm) | mean | std err | lower | upper | p-value |
| --- | --- | --- | --- | --- | --- |
| 15 | 16.7526 | 0.9435 | 13.9356 | 19.5696 | 5.507E-10 |
| 25 | 11.1887 | 1.0280 | 8.0376 | 14.3398 | 4.162E-06 |
| 40 | 16.3841 | 0.3591 | 15.3050 | 17.4632 | -3.331E-15 |
| 60 | 11.4483 | 0.3409 | 10.3665 | 12.5300 | 8.578E-10 |
| 90 | 9.3066 | 0.3342 | 8.2413 | 10.3718 | 7.867E-09 |

Table 7. Games-Howell post hoc test for mean differences in liquid retained (percent of camera) between the complex and simple chamber models of *Damesites* (with hydrophilic coating). Lower and upper 95% confidence intervals and p-values are reported at a confidence level (α) of 0.05. The number of measurements (n) for each model is 10. Sizes are measured as whorl heights (Wh; dorsoventral length of the camera).

| Wh (mm) | mean | std err | lower | upper | p-value |
| --- | --- | --- | --- | --- | --- |
| 15 | 7.7455 | 1.1275 | 4.3374 | 11.1535 | 2.287E-04 |
| 25 | 9.4450 | 0.3590 | 8.3719 | 10.5182 | 1.608E-12 |
| 40 | 9.9415 | 0.2354 | 9.2420 | 10.6410 | 1.033E-14 |
| 60 | 14.0036 | 0.2167 | 13.3155 | 14.6918 | 3.062E-11 |
| 90 | 12.4796 | 0.1410 | 12.0378 | 12.9214 | -4.579E-12 |

Table 8. Mean and standard deviation (std. dev.) of the percentage liquid retained in the chamber models of *Menuites oralensis*. C = complex suture, S = smoothed suture, with following numbers indicating the number of smoothing iterations. The number of measurements for each model type (n) is 10.

| *Menuites* | No coating | | Hydrophilic coating | |
| --- | --- | --- | --- | --- |
| group (15 mm) | mean | std. dev. | mean | std. dev. |
| C | 11.4778 | 0.9446 | 18.4062 | 0.8515 |
| S1 | 11.3449 | 1.6654 | 17.6247 | 1.1130 |
| S2 | 10.1044 | 2.3243 | 15.4414 | 1.8967 |
| S3 | 10.4450 | 0.8735 | 15.5151 | 2.4737 |
| group (25 mm) | mean | std. dev. | mean | std. dev. |
| C | 9.6274 | 1.1206 | 13.7235 | 0.5563 |
| S1 | 8.8414 | 0.9284 | 12.3696 | 0.5527 |
| S2 | 8.7541 | 0.6203 | 10.4625 | 0.6380 |
| S3 | 7.2570 | 0.6675 | 9.3323 | 0.9023 |
| group (40 mm) | mean | std. dev. | mean | std. dev. |
| C | 8.0943 | 0.3378 | 10.5552 | 0.3278 |
| S1 | 7.7081 | 0.5170 | 9.6204 | 0.4056 |
| S2 | 7.1043 | 0.1499 | 9.1675 | 0.1656 |
| S3 | 3.7895 | 0.5458 | 6.0402 | 0.4222 |
| group (60 mm) | mean | std. dev. | mean | std. dev. |
| C | 6.6712 | 0.4855 | 9.2671 | 0.2841 |
| S1 | 5.9809 | 0.2820 | 7.3209 | 0.2128 |
| S2 | 4.8828 | 0.1864 | 5.7285 | 0.1468 |
| S3 | 1.6809 | 0.2186 | 5.2195 | 0.1905 |
| group (90 mm) | mean | std. dev. | mean | std. dev. |
| C | 4.3654 | 0.1169 | 6.6426 | 0.2640 |
| S1 | 4.0815 | 0.1479 | 5.2765 | 0.1219 |
| S2 | 2.3113 | 0.1700 | 4.2409 | 0.0643 |
| S3 | 0.7710 | 0.1050 | 1.6094 | 0.0961 |

Table 9. Games-Howell post hoc test for mean differences in liquid retained (percent of camera) between the chamber models of *Menuites oralensis* (with no coating). C = complex suture, S = smoothed suture, with following numbers indicating the iteration of smoothing. Lower and upper 95% confidence intervals and p-values are reported at a confidence level (α) of 0.05. The number of measurements (n) for each model is 10. Highlighted p-values are greater than 0.05 and are deemed statistically indistinguishable.

| group 1  (15 mm) | group 2  (15 mm) | mean | std err | lower | upper | p-value |
| --- | --- | --- | --- | --- | --- | --- |
| C | S1 | 0.1328 | 0.4281 | -1.6233 | 1.8890 | 0.9961 |
| C | S2 | 1.3734 | 0.5610 | -0.9855 | 3.7322 | 0.3511 |
| C | S3 | 1.0327 | 0.2877 | -0.1179 | 2.1833 | 0.0876 |
| S1 | S2 | 1.2405 | 0.6394 | -1.3409 | 3.8220 | 0.5332 |
| S1 | S3 | 0.8999 | 0.4205 | -0.8352 | 2.6350 | 0.4568 |
| S2 | S3 | 0.3407 | 0.5552 | -2.0061 | 2.6874 | 0.9714 |
| group 1 (25 mm) | group 2 (25 mm) | mean | std err | lower | upper | p-value |
| C | S1 | 0.7860 | 0.3254 | -0.5191 | 2.0910 | 0.3494 |
| C | S2 | 0.8733 | 0.2864 | -0.3037 | 2.0503 | 0.1835 |
| C | S3 | 2.3703 | 0.2917 | 1.1783 | 3.5624 | 2.204E-04 |
| S1 | S2 | 0.0873 | 0.2497 | -0.9250 | 1.0997 | 0.9945 |
| S1 | S3 | 1.5844 | 0.2557 | 0.5523 | 2.6165 | 2.259E-03 |
| S2 | S3 | 1.4970 | 0.2038 | 0.6822 | 2.3119 | 3.312E-04 |
| group 1 (40 mm) | group 2 (40 mm) | mean | std err | lower | upper | p-value |
| C | S1 | 0.3862 | 0.1381 | -0.1746 | 0.9469 | 0.2383 |
| C | S2 | 0.9900 | 0.0826 | 0.6447 | 1.3353 | 8.624E-06 |
| C | S3 | 4.3048 | 0.1435 | 3.7199 | 4.8897 | 7.005E-12 |
| S1 | S2 | 0.6038 | 0.1204 | 0.0875 | 1.1202 | 2.160E-02 |
| S1 | S3 | 3.9186 | 0.1681 | 3.2465 | 4.5907 | 1.589E-11 |
| S2 | S3 | 3.3148 | 0.1266 | 2.7705 | 3.8590 | 1.472E-08 |
| group 1  (60 mm) | group 2 (60 mm) | mean | std err | lower | upper | p-value |
| C | S1 | 0.6903 | 0.1255 | 0.1763 | 1.2044 | 7.504E-03 |
| C | S2 | 1.7884 | 0.1163 | 1.2976 | 2.2793 | 1.029E-06 |
| C | S3 | 4.9904 | 0.1191 | 4.4934 | 5.4873 | 3.269E-12 |
| S1 | S2 | 1.0981 | 0.0756 | 0.7914 | 1.4047 | 1.329E-07 |
| S1 | S3 | 4.3000 | 0.0798 | 3.9792 | 4.6209 | 4.763E-14 |
| S2 | S3 | 3.2019 | 0.0642 | 2.9445 | 3.4593 | 1.987E-14 |
| group 1 (90 mm) | group 2  (90 mm) | mean | std err | lower | upper | p-value |
| C | S1 | 0.2839 | 0.0422 | 0.1145 | 0.4533 | 9.309E-04 |
| C | S2 | 2.0542 | 0.0461 | 1.8674 | 2.2409 | 8.882E-15 |
| C | S3 | 3.5944 | 0.0351 | 3.4538 | 3.7350 | 1.354E-14 |
| S1 | S2 | 1.7702 | 0.0504 | 1.5685 | 1.9720 | 3.841E-14 |
| S1 | S3 | 3.3105 | 0.0406 | 3.1466 | 3.4743 | 6.217E-15 |
| S2 | S3 | 1.5403 | 0.0447 | 1.3581 | 1.7224 | 1.063E-12 |

Table 10. Games-Howell post hoc test for mean differences in liquid retained (percent of camera) between the chamber models of *Menuites oralensis* (with hydrophilic coating). C = complex suture, S = smoothed suture, with following numbers indicating the iteration of smoothing. Lower and upper 95% confidence intervals and p-values are reported at a confidence level (α) of 0.05. The number of measurements (n) for each model is 10. Highlighted p-values are greater than 0.05 and are deemed statistically indistinguishable.

| group 1  (15 mm) | group 2  (15 mm) | mean | std err | lower | upper | p-value |
| --- | --- | --- | --- | --- | --- | --- |
| C | S1 | 0.7815 | 0.3134 | -0.4794 | 2.0423 | 0.3241 |
| C | S2 | 2.9647 | 0.4649 | 1.0240 | 4.9055 | 3.151E-03 |
| C | S3 | 2.8911 | 0.5850 | 0.4051 | 5.3770 | 2.197E-02 |
| S1 | S2 | 2.1833 | 0.4917 | 0.1713 | 4.1952 | 3.146E-02 |
| S1 | S3 | 2.1096 | 0.6065 | -0.4221 | 4.6413 | 0.1161 |
| S2 | S3 | 0.0737 | 0.6970 | -2.7307 | 2.8780 | 0.9998 |
| group 1 (25 mm) | group 2 (25 mm) | mean | std err | lower | upper | p-value |
| C | S1 | 1.3539 | 0.1754 | 0.6530 | 2.0548 | 1.870E-04 |
| C | S2 | 3.2610 | 0.1893 | 2.5031 | 4.0189 | 2.817E-09 |
| C | S3 | 4.3912 | 0.2370 | 3.4250 | 5.3574 | 7.284E-09 |
| S1 | S2 | 1.9071 | 0.1887 | 1.1512 | 2.6630 | 7.330E-06 |
| S1 | S3 | 3.0373 | 0.2366 | 2.0723 | 4.0023 | 1.002E-06 |
| S2 | S3 | 1.1302 | 0.2471 | 0.1318 | 2.1286 | 2.383E-02 |
| group 1 (40 mm) | group 2 (40 mm) | mean | std err | lower | upper | p-value |
| C | S1 | 0.9349 | 0.1166 | 0.4668 | 1.4029 | 1.413E-04 |
| C | S2 | 1.3877 | 0.0821 | 1.0479 | 1.7275 | 9.192E-08 |
| C | S3 | 4.5150 | 0.1195 | 4.0344 | 4.9956 | 6.373E-14 |
| S1 | S2 | 0.4528 | 0.0980 | 0.0411 | 0.8646 | 2.989E-02 |
| S1 | S3 | 3.5801 | 0.1309 | 3.0568 | 4.1035 | 1.044E-12 |
| S2 | S3 | 3.1273 | 0.1014 | 2.6998 | 3.5547 | 4.357E-10 |
| group 1  (60 mm) | group 2 (60 mm) | mean | std err | lower | upper | p-value |
| C | S1 | 1.9461 | 0.0794 | 1.6264 | 2.2658 | 2.420E-11 |
| C | S2 | 3.5386 | 0.0715 | 3.2433 | 3.8340 | 1.518E-13 |
| C | S3 | 4.0476 | 0.0765 | 3.7375 | 4.3576 | 9.992E-16 |
| S1 | S2 | 1.5925 | 0.0578 | 1.3585 | 1.8265 | 8.118E-12 |
| S1 | S3 | 2.1014 | 0.0639 | 1.8458 | 2.3570 | 6.550E-14 |
| S2 | S3 | 0.5089 | 0.0538 | 0.2926 | 0.7252 | 2.129E-05 |
| group 1 (90 mm) | group 2  (90 mm) | mean | std err | lower | upper | p-value |
| C | S1 | 1.3661 | 0.0650 | 1.0952 | 1.6370 | 1.169E-08 |
| C | S2 | 2.4017 | 0.0608 | 2.1391 | 2.6643 | 2.811E-10 |
| C | S3 | 5.0332 | 0.0628 | 4.7672 | 5.2993 | -1.622E-12 |
| S1 | S2 | 1.0356 | 0.0308 | 0.9085 | 1.1627 | 1.023E-11 |
| S1 | S3 | 3.6671 | 0.0347 | 3.5277 | 3.8066 | 4.108E-14 |
| S2 | S3 | 2.6315 | 0.0259 | 2.5267 | 2.7364 | 1.110E-15 |

Table 11. Means and standard deviations (std. dev.) of percent liquid retained in a single camera of a modern *Nautilus pompilius* (terminal chamber; whorl height of 62 mm). Ten measurements were recorded under three separate conditions: 1) with the original, desiccated pellicle lining the chamber (Original), 2) with most of the pellicle scraped away (W/O pellicle), and 3) with micro-dispersed oxidized cellulose lining the chamber (With MDOC).

| *group* | *mean* | *std. dev.* |
| --- | --- | --- |
| Original | 3.4529 | 0.1772 |
| W/O pellicle | 3.1246 | 0.3759 |
| With MDOC | 11.8932 | 0.7573 |

Table 12. Games-Howell post hoc test for mean differences in liquid retained (percent of camera) between the *Nautilus pompilius* chamber experiments. Ten measurements were recorded under three separate conditions: 1) with the original, desiccated pellicle lining the chamber (Original), 2) with most of the pellicle scraped away (W/O pellicle), and 3) with micro-dispersed oxidized cellulose lining the chamber (With MDOC). Lower and upper 95% confidence intervals and p-values are reported at a confidence level (α) of 0.05. Highlighted p-values are greater than 0.05 and are deemed statistically indistinguishable.

| group 1 | group 2 | mean | std err | lower | upper | p-value |
| --- | --- | --- | --- | --- | --- | --- |
| Original | W/O pellicle | 0.3283 | 0.0929 | -0.0193 | 0.6760 | 0.0650 |
| Original | With MDOC | 8.4402 | 0.1739 | 7.7658 | 9.1147 | 2.199E-11 |
| W/O pellicle | With MDOC | 8.7686 | 0.1890 | 8.0639 | 9.4733 | 3.137E-13 |

Dataset 1 (separate file). Virtual, 3D models for the capillary retention experiments are stored in STL format in an online repository^4^ (http://doi.org/10.5281/zenodo.4308813). This dataset contains 1) cylindrical models containing single septa, and 2) individual camerae of *Damesites* sp. and *Menuites oralensis*. The chamber models also include versions of each species with their sutures successively smoothed by Laplacian algorithms in Blender (5). Because these virtual models are scalable and the total file size must be managed, the uploaded chamber models are of a single size (60 mm whorl height; dorsoventral length). In order to recreate the cameral liquid retention experiments, these models must be subtracted from arbitrary bounding volumes (depending on the desired scale) in the program Blender^5^ or Netfabb^6^.

**SI References**

1. Ward, P.D. Cameral liquid in Nautilus and ammonites. Paleobiology **5**, 40–49 (1979).
2. Hoffmann, R., Lemanis, R., Naglik, C. & Klug, C. Ammonoid Buoyancy in *Ammonoid Paleobiology: from anatomy to ecology* (eds. Klug, C., Korn, D., De Baets, K., Kruta, I. & Mapes, R.H.) Topics in Geobiology 44, 611–648 (Springer, 2015).
3. Klug, C., Hoffmann, R. Ammonoid septa and sutures *in Ammonoid Paleobiology: from anatomy to ecology* (eds. Klug, C., Korn, D., De Baets, K., Kruta, I. & Mapes, R.H.) Topics in Geobiology 44, 45–90 (Springer, 2015).
4. Peterman, D.J. Septa and camerae models for cameral liquid retention experiments [Data set]. Zenodo. Available at http://doi.org/10.5281/zenodo.4308813. Deposited December 7, 2020.
5. Blender Online Community. Blender, a 3D modelling and rendering package. Blender Institute, Amsterdam. http://www.blender.org, (2017).
6. Autodesk Inc., Netfabb 2017.3. Autodesk Inc., San Rafael, CA (2017).
